# A Missense Mutation in a Highly Conserved Alternate Exon of Dynamin-1 Causes Epilepsy in Fitful Mice

**DOI:** 10.1371/journal.pgen.1001046

**Published:** 2010-08-05

**Authors:** Rebecca M. Boumil, Verity A. Letts, Monica C. Roberts, Christine Lenz, Connie L. Mahaffey, Zhong-wei Zhang, Tobias Moser, Wayne N. Frankel

**Affiliations:** 1 The Jackson Laboratory, Bar Harbor, Maine, United States of America; 2 InnerEarLab, Department of Otolaryngology and Center for Molecular Physiology of the Brain, Bernstein Center for Computational Neuroscience, University of Göttingen, Göttingen, Germany; Stanford University School of Medicine, United States of America

## Abstract

Dynamin-1 (*Dnm1*) encodes a large multimeric GTPase necessary for activity-dependent membrane recycling in neurons, including synaptic vesicle endocytosis. Mice heterozygous for a novel spontaneous *Dnm1* mutation—fitful—experience recurrent seizures, and homozygotes have more debilitating, often lethal seizures in addition to severe ataxia and neurosensory deficits. Fitful is a missense mutation in an exon that defines the DNM1a isoform, leaving intact the alternatively spliced exon that encodes DNM1b. The expression of the corresponding alternate transcripts is developmentally regulated, with DNM1b expression highest during early neuronal development and DNM1a expression increasing postnatally with synaptic maturation. Mutant DNM1a does not efficiently self-assemble into higher order complexes known to be necessary for proper dynamin function, and it also interferes with endocytic recycling in cell culture. In mice, the mutation results in defective synaptic transmission characterized by a slower recovery from depression after trains of stimulation. The DNM1a and DNM1b isoform pair is highly conserved in vertebrate evolution, whereas invertebrates have only one isoform. We speculate that the emergence of more specialized forms of DNM1 may be important in organisms with complex neuronal function.

## Author Summary

Epilepsy, a group of chronic disorders characterized by recurrent seizures, results from abnormal, synchronized neuronal activity in the brain. The mouse represents a powerful system to study novel mutations that model neurological disease, including epilepsy. Here we describe a new mouse mutation (“fitful”) in the gene encoding dynamin-1. Fitful mice have recurrent seizures and other neurological defects, including impaired hearing. Dynamin-1 is very well studied, but has yet to be linked to neurological disease. Dynamin-1 is a large multimeric enzyme that functions in membrane fission, primarily of vesicles after they release neurotransmitter at neuronal synapses. Fitful occurs in the region of dynamin-1 that is important for self-assembly of single dynamin subunits into the multimers required for enzymatic function. We show that fitful interferes with dynamin-1 self-assembly and with endocytosis. Moreover, the mutation resides in one of two alternate forms of dynamin-1 and affects what may be a necessary shift during brain development, with the expression of the mutated form being higher after maturation in fitful mice. This particular genetic specialization is unique to vertebrate dynamin. We speculate that specialized forms of dynamin-1 are important for modifying the self-assembly process to meet the demands complex brain activity in higher organisms.

## Introduction

Epilepsy affects about 1% of the population and approximately 30% of cases are idiopathic, with no obvious explanation such as head injury, stroke, lesions or tumors [1]. Genetic factors are thought to lie behind idiopathic epilepsy [1]. Several human epilepsy genes have been identified [2], [3] but most genes for common idiopathic epilepsy remain unknown, in part due to the complex genetics. Employing a forward genetics approach, we have studied the mutant “ fitful” mouse which exhibits spontaneous limbic and generalized tonic-clonic seizures upon routine handling, due to a spontaneous mutation in the gene encoding dynamin-1. Heterozygous fitful mice develop epilepsy by two to three months of age, but are otherwise outwardly normal. Homozygous mice have a more severe neurological phenotype at a much younger age - three weeks - including ataxia, hearing and vision defects and lethal seizures.

Dynamin-1 belongs to a family of large GTPases that function in endocytosis, vesicle scission, membrane recycling, organelle division, cytokinesis and antiviral activity [4]–[10]. In mammals, there are three dynamin genes (*Dnm1*, *Dnm2*, and *Dnm3*) each of which undergoes complex alternative splicing resulting in over 25 dynamin isoforms. *Dnm2* is expressed in all tissues [11]. *Dnm1* is expressed only in the brain, localizing to the presynaptic terminal [12], [13]. *Dnm3* is expressed in the brain (where it is associated with the postsynaptic compartment) and the testes [12], [14]. Flies carrying mutant temperature-sensitive alleles of *shibire*, the Drosophila homolog of dynamin, exhibit paralysis at the restrictive temperature that is due to depletion of synaptic vesicles in a use-dependent manner [15].

Dynamin-1 has an established role in endocytic vesicle fission from the plasma membrane [16] and its expression is upregulated in the brain during postnatal development, concomitant with synaptogenesis. In primary neuronal culture, the expression and protein levels of dynamin-1 increase steadily in conjunction with the formation of neurites over time in culture, peaking as synapse formation occurs [9], [12], [17]. This expression pattern mimics that of other synaptic vesicle proteins such as synaptophysin and suggests a critical role for dynamin-1 in synaptic vesicle recycling based on its developmental expression pattern as well as localization to presynaptic compartments and known function in membrane recycling and endocytosis.

Dynamin molecules assemble into tetrameric structures that hydrolyse GTP to scission vesicle membrane, including synaptic vesicles which recycle after neurotransmitter release. Dynamin monomers are 100KD polypeptides containing 5 functional domains: a GTPase domain that binds and hydrolyses GTP, a middle domain that is involved in oligomerization, a GTPase effector domain (GED) that is involved in oligomerization and stimulation of GTPase activity, and plextrin-homology (PH) and proline-rich (PRD) domains that mediate lipid- and protein-protein interactions. In fitful mice, a single nucleotide change results in an amino acid substitution in the highly conserved coding region of the middle domain of the protein. This domain is involved in the dimerization and higher order assembly of the dynamin tetramers [18]–[20]. Interestingly, mutations in the corresponding middle domain of dynamin-2 underlie human disease such as autosomal dominant centronuclear myopathy [21] and a canine mutation in *Dnm1* located at the GTPase/middle domain border results in an exercise induced collapse disorder in Labrador retrievers [22].

Mice that completely lack *Dnm1* survive into the second week of life demonstrating that *Dnm1* is not necessary for embryonic development or for perinatal synaptic transmission [9]. In *Dnm1* null mice, inhibitory neurons are more sensitive to the loss of *Dnm1* and experience endocytic defects [9], [10], but neither the homozygous nor the heterozygous *Dnm1* null mice have seizures [9]. In cortical neuron cultures established from *Dnm1* null mice, inhibitory neurons are shown to be the most sensitive to loss of *Dnm1*, acquiring a large build up of endocytic intermediates during spontaneous network activity [10]. Silencing transmission relieves the endocytic defect - suggesting that the high intrinsic level of tonic activity of these cortical inhibitory neurons makes them more vulnerable to the lack of *Dnm1* [10].

*Dnm1*, as well as *Dnm2* and *Dnm3*, is alternatively spliced at two locations in the transcript resulting in the possibility of nine *Dnm1* isoforms. In this study, we show that the fitful mutation in *Dnm1* interferes with the normal expression of the first alternatively spliced region during postnatal development. This region is located within the middle domain required for oligomerization, a process that is affected in fitful mutants.

Given that *Dnm1* is essential for synaptic vesicle endocytosis, fitful mice are likely to have a lack of sufficient vesicles for extended synaptic transmission. This may be particularly problematic for the recycling of synaptic vesicles at tonically firing inhibitory synapses in light of findings in dynamin-1 null mice [9], [10]. Disruption of this process would consequently upset the balance between inhibition and excitation resulting in abnormal propagation of neuronal excitability and recurrent seizures. Previous studies have extensively examined the function and mechanisms of action of *Dnm1*, but this study is the first to identify a *Dnm1* mutation that leads to epilepsy. Furthermore, our study suggests that there are distinct roles for the alternatively spliced *Dnm1* isoforms which have not previously been examined *in vivo*. The fitful mouse model provides a unique resource for understanding the role of the *Dnm1* isoforms in brain as well as for studying the frequency-dependent weakening of synaptic inhibition as has been seen in other genetic models of generalized epilepsy, such as *SCN1A* [23], [24].

## Results

### The “fitful” mouse as a model of generalized idiopathic epilepsy

**Figure 1:**
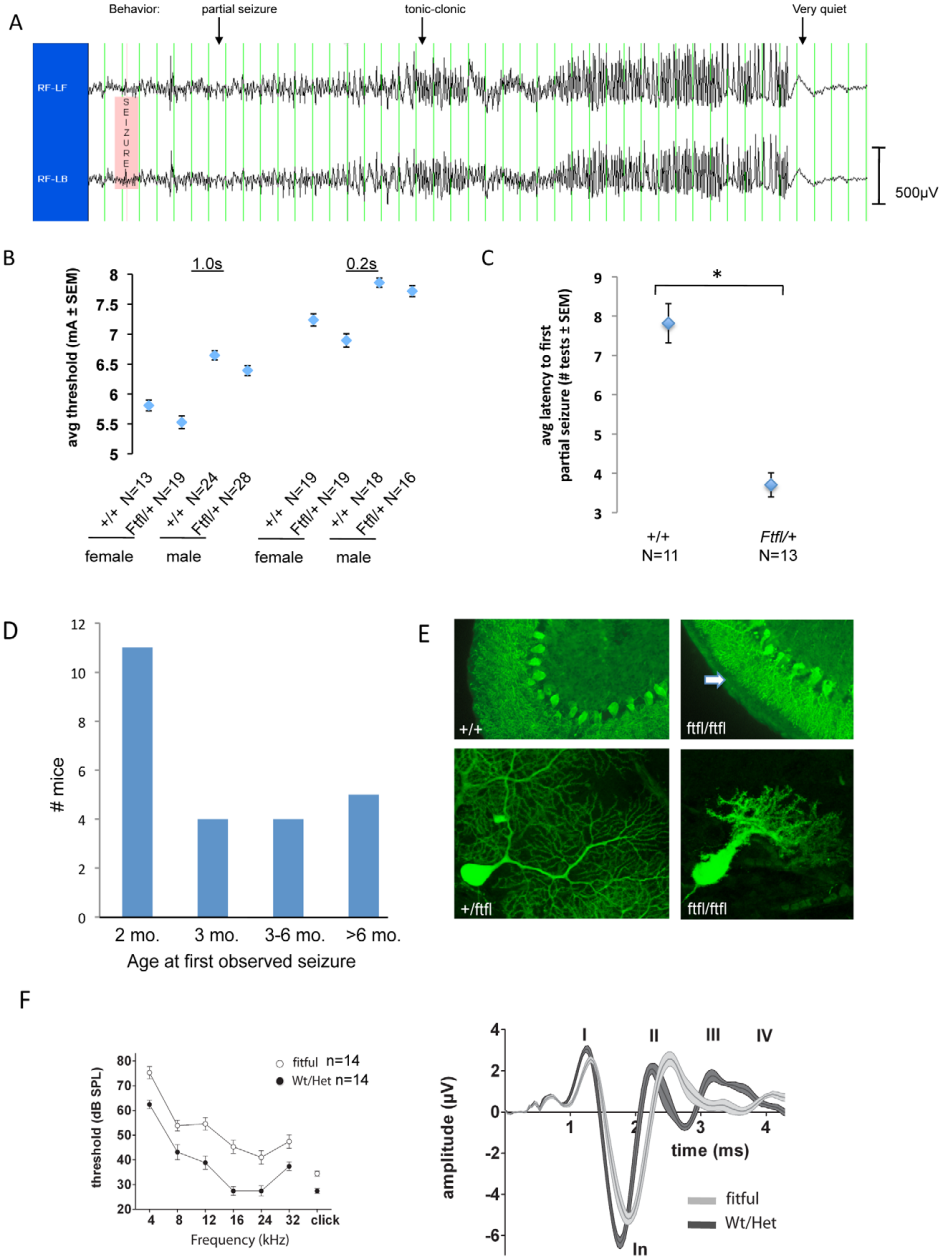
Fitful mice have seizures and neurosensory defects. (A) Differential traces from EEG recording of fitful heterozygotes. Behavioral events associated with the EEG are shown. Vertical lines are 1s time markings; each channel is approximately 500uV high. (B) Average electroconvulsive thresholds to the minimal forebrain clonic seizure endpoint of B6-Ftfl/+ and B6-+/+ mice at two different stimulus durations (1.0s – left; 0.2s – right). Female and male mice are shown separately. The combined data showed that this modest difference between genotypes in acute seizure threshold was statistically significant (Student's |t|-test, p<0.001). (C) Average latency (# of daily tests) to the first kindled partial seizure following sequential stimulation in B6-+/+ and B6-Ftfl/+ male mice (*Student's |t|-test, p<0.0001). (D) Graph showing the age of onset of observed (behavioral tonic-clonic) seizures in fitful heterozygous mice in the B6 or FVB background. (E) Purkinje cell defect in fitful cerebellum. The upper panels show calbindin antibody staining of wildtype and fitful P15 cerebellum. Notice that the Purkinje cell dendrites are stunted (arrow) in the mutant as compared to wildtype and the soma are less ordered. The lower panels show B6.FVB fitful homozygous and heterozygous Purkinje cells expressing GFP at P17. (F) Auditory brainstem response in fitful and wildtype mice. Left panel: ABR audiograms with average thresholds ± SEM of fitful mouse mutants (open symbols, −/−, n = 14) and their wildtype (n = 5; +/+) and heterozygote (n = 9; +/−) littermates (closed symbols). Right panel: average ABR waveforms (±SEM) of fitful (light grey, n = 9) and wildtype/heterozygous mice (dark grey, n = 9) in response to click stimuli (86 dB, peak equivalent). Latin numbers denominate the ABR peaks.

The fitful mutation (allele symbol: *Ftfl*) arose spontaneously in C57BL/6J (B6) mice and was identified initially by the occurrence of recurrent, non-lethal seizures. The overall phenotype of *Ftfl* is semidominant. Heterozygotes develop partial and generalized tonic-clonic seizures upon routine handling from approximately two to three months of age (Figure 1A and 1D), but otherwise appear normal and have a lifespan similar to wildtype littermates. Heterozygotes have a modest reduction in seizure threshold to an acute electrical stimulus, about 0.25 mA lower than controls (Figure 1B). Although this effect is statistically significant, it is smaller than the difference (>0.5 mA) we have reported in other seizure-prone mice on the B6 background - some of which go on to develop epilepsy (*Brunol4^Ff^*; [25]), and others that do not (*Szt2*;[26]). Nevertheless, heterozygous fitful mice develop kindled seizures much more readily than wildtype mice in response to repeated electrical stimuli, suggesting that they are more epileptogenic (Figure 1C).

Homozygous fitful mutants have more severe phenotypes, including ataxia and spontaneous convulsive seizures that usually result in death before weaning age (see Video S1). Homozygous pups are born with the expected Mendelian genotype ratio, but only survive into the second or third week of life depending on strain background. Mutant pups are viable and indistinguishable from their wildtype littermates at birth and for the first week of life. At approximately post-natal day 12 (P12), the mutants become discernible from wildtype as they develop a progressive ataxia characterized by an abnormal and uncoordinated stance and gait. Homozygotes show tonic-clonic seizures at P14–P16. A seizure episode typically lasts 30 seconds to one minute and is immediately followed by a clear diminishment in health and movement. Typically, mutants die before P18 from either a lethal seizure or lack of nourishment brought on by continued weakening of physical movement. Mutant pups that receive nutritional supplementation tend to survive about one to two days longer.

Routine histology showed no obvious brain abnormalities in homozygous mice (data not shown). However, when examined by immunofluorescence, Purkinje cell dendritic trees were markedly smaller in all homozygous mice examined at P17 (Figure 1E). In mutants, Purkinje cell dendrites were polydendritic and the size of the dendritic arbor and degree of branching was reduced compared to wildtype (Figure 1E, bottom panels). The wildtype Purkinje cell layer had more regular somas that are ordered in structure and dendrites that are noticeably smoother than the mutants that showed spiny dendrites along with a disorderly arrangement of soma.

### Hearing impairment in fitful homozygotes

We suspected that the homozygotes might have neurosensory defects. Given their lethality at three weeks of age, hearing was the most practical sense to test in detail. To examine hearing, we evaluated the auditory brainstem response (ABR) of P14–17 fitful homozygous mice and compared with wildtype and heterozygous littermates. Fitful homozygotes display a modest ABR threshold increase, approximately 15 dB, across all sound frequencies tested (Figure 1F, left panel). We also found that the latencies of most ABR peaks were progressively prolonged (Figure 1F, right panel) and the amplitude of some of the ABR peaks was reduced in the mutants. The first peak (peak I) was reduced and delayed reflecting impaired function of the inner hair cell-afferent synapse and a reduction in synchronous spiral ganglion activation [27], [28]. To rule out a defect in outer hair cell function, we measured distortion product otoacoustic emissions. This showed intact outer hair cell function in fitful homozygotes (Figure S1). Thus, the hearing impairment is most likely due to a deficit in sound coding at the inner hair cell synapse that could be due to a lack of a rapid resupply of readily-releasable vesicles [29].

### The *Ftfl* mutation resides in an alternative exon of *Dnm1*

**Figure 2:**
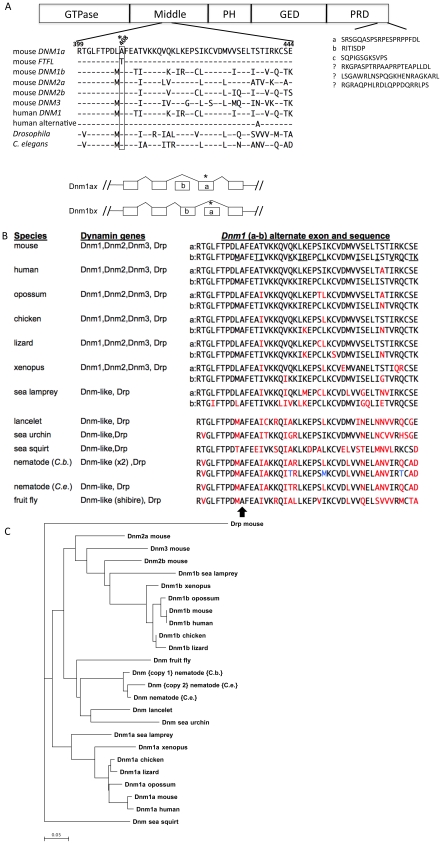
Conserved protein sequence alignment of Dynamins. (A) Conserved protein sequence alignment. The upper diagram shows DNM1 sequence conservation among various species and the location of the fitful mutation within the middle domain of the protein. The first splice region (amino acids 399–444) shows the location of the fitful mutation (*408) and the conservation within mammalian, fly and worm dynamins. Notice that the fly and worm orthologs do not have splice variants in this region. The second alternative splicing region is also shown at the end of the PRD domain. GTPase, GTPase domain; Middle, middle domain; PH, pleckstrin homology domain; GED, GTPase effector domain; PRD, proline rich domain. The bottom diagram depicts the mutually exclusive alternative splicing of the *Dnm1a* and *Dnm1b* isoforms. (B) Putative orthologues of mouse *Dnm1*, *Dnm2*, *Dnm3* and *Drp* (dynamin related protein – official mouse gene symbol, *Dnm1l*) from different species. For simplicity, the same generic gene symbol is used for all; for simpler eukaryotes, the symbol Dnm refers to their orthologue(s) most like the mammalian *Dnm1*, *Dnm2* or *Dnm3*. The known or predicted alternate sequences corresponding to mouse *Dnm1* exon 10 are also shown. The underlined amino acid symbols show where mouse Dnm1b differs from Dnm1a. The arrow at the bottom shows the highly conserved alanine residue that is mutated to threonine in the mouse *Dnm1^Ftfl^* allele. Residues colored in red show amino acid substitutions with respect to mouse *Dnm1*; for nematode (C.b. - *C. briggsae*; C.e. – *c.elegans*), differences between the two Dnm peptides are shown in blue. Peptide sequences were obtained as follows: mouse Dnm1a: GenBank AAA37318, Dnm1b : GenBank EDL08539; human Dnm1a: GenBank AAA02804, Dnm1b: GenBank AAA02803; nematode [48] GenBank AAB72228; fruit fly (*D. melanogaster*) EMBL CAA42068. The remaining predicted sequences were obtained from analysis using the USCS Genome Browser and draft genome sequence assemblies from the following respective genome centers: chicken (G. *gallus*), sea lamprey (*P. marinus*) and nematode (*C. briggsae*) - Genome Sequencing Center, Washington University School of Medicine; opposum (*M. domestica*) and lizard (*A. carolinensis*), The Broad Institute; xenopus (X. *tropicalis*), lancelet (*B. floridae*), and sea squirt (*C. intestinalis*) - DoE Joint Genome Institute; sea urchin (*S. purpuratus*) - Baylor College of Medicine Human Genome Sequencing Center. The dynamin gene composition from sea lamprey was inferred from sequence alignment of draft 5.9-fold genome sequence (accessed via the UCSC browser - genome.ucsc.edu). When used as query, the respective mouse *Dnm1*, *Dnm2* and *Dnm3* peptide sequences corresponding to the assembly domain region each yielded significant alignments with only a single, approximately 20kb contig (Contig16000). This contig contained seven Dnm-like exons, with appropriate splice site recognition motifs, that was co-linear with mouse *Dnm1* exons 8–13, including exons corresponding to Dnm1b (exon 10b) and Dnm1a (exon 10a) in the expected 5′-3′ arrangement. (C) Neighbor-joining best tree of dynamin peptides. Proportional number of differences is estimated at the bottom. Note the closer relationship between the two isoforms from sea lamprey and the respective Dnm1a and Dnm1b branches from more complex vertebrates. Also note the closer relationship between Dnm2 isoforms and invertebrate dynamin.

Whole genome scans were done to map both the dominant seizure phenotype, and the recessive ataxia phenotype, to Chromosome 2. Further fine mapping narrowed the region to two Mb including the *Dnm1* gene and several other candidate genes with high expression in the brain: *Mapkap1*, *Fibcd1*, *Ppapdc1*, and *Stxbp1*. Expression analysis of these genes did not reveal any differences between wildtype and mutant brain RNA, and further cDNA sequencing of *Stxbp1* specifically revealed no mutations. In *Dnm1* a single nucleotide change was discovered by sequence analysis of mutant cDNA, revealing a G-to-A nucleotide mutation in the first of two alternatively spliced regions (Figure 2A).

*Dnm1* encodes several isoform variants resulting from alternative mRNA splicing events at two regions: the middle domain, which contains the fitful mutation, and the C-terminal PRD domain. Splicing results in either DNM1a*x* or DNM1b*x*, where a or b are the middle domain splice variants and *x* is any of the three alternatively spliced terminal exons. The middle domain variant differs only by a peptide encoded by two tandemly arranged exons, with 14 residues varying within a 46 amino acid region (Figure 2A). This particular alternative exon pair of *Dnm1* and *Dnm2* is conserved in all vertebrates including mice (e.g. *Dnm1a*x and *Dnm1b*x) and humans (*Dnm1* and *Dnm1* “alternative”), but it is not present in *Dnm3* nor is it in invertebrate dynamin (Figure 2A–2C; [30], [31], reviewed in [32]; see Figure 2B legend for more details). The fitful missense mutation results in an alanine to threonine substitution at amino acid 408, an evolutionarily conserved residue (Figure 2A and 2B). Noticeably, fitful only affects the *Dnm1ax* isoform sequences; the “a” exon is spliced out in the *Dnm1bx* forms, resulting in potentially all three *Dnm1ax* transcripts being altered.

**Figure 3:**
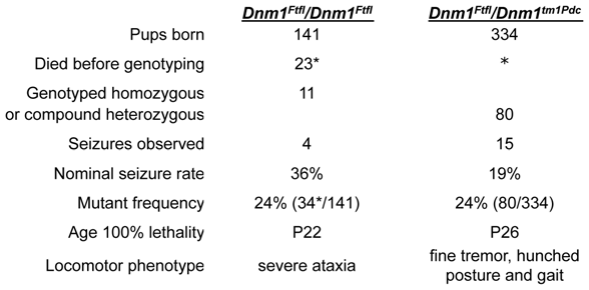
Fitful and compound heterozygous *Dnm1* seizure and locomotor phenotypes. Shown is the frequency of fitful homozygous (*Dnm1^Ftfl^/Dnm1^Ftfl^*) and compound heterozygous (*Dnm1^Ftfl^/Dnm1^tm1Pdc^*) mutant mice and their respective seizure incidence and locomotor phenotypes. The fitful homozygotes shown were from fully informative matings from the mapping cross used to map the recessive phenotype; the compound heterozygotes were from crosses between single heterozygotes from respective FVB-fitful and B6-null colonies. The asterisk is used to indicate that the latter population was observed almost daily from P12 through demise (P16–P23), whereas the former were observed at weaning age only – suggesting that death occurred in these mutants between P12 and P21.

To provide genetic confirmation that the *Ftfl* phenotype is caused by the missense mutation in *Dnm1*, we crossed heterozygous *Dnm1* ^Ftfl^ mice to mice heterozygous for the null mutation (*Dnm1* ^tm1Pdc^; [9]). Compound heterozygous *Dnm1* ^tm1Pdc^/*Dnm1* ^Ftfl^ pups were born very near to the expected Mendelian ratio of 1∶4 (Figure 3). We observed 80 compound heterozygotes out of 46 litters having a total of 334 pups. The average litter size was 6–8 pups depending on background strain. The phenotype of the resulting *Dnm1* ^tm1Pdc^/*Dnm1* ^Ftfl^ compound heterozygous mice was in many ways similar to that of *Ftfl* homozygotes, including seizures by the third week of life and death before weaning. We observed 15 compound heterozygous mice to have seizures during their shortened lifespan, while no such seizures were observed in wildtype, or deleted or fitful heterozygous littermates at this age (Figure 3). There were four confirmed deaths due to seizures, with three more suspected by virtue of finding carcasses with hindlimb or forelimb tonic extension. Although the onset and appearance of lethal seizures was very similar between fitful homozygotes and compound heterozygotes, these two classes had different locomotor phenotypes; the compound heterozygotes were not ataxic, although they had a slight tremor and hunched appearance that continually increased in severity. Together with the mapping and mutation analysis, these results provide strong evidence that fitful is an allele of *Dnm1*. They also reinforce the suggestion that wildtype *Dnm1* is necessary for normal postnatal neurodevelopment.

### Fitful alters the developmental expression of *Dnm1* isoforms at RNA and protein level

**Figure 4:**
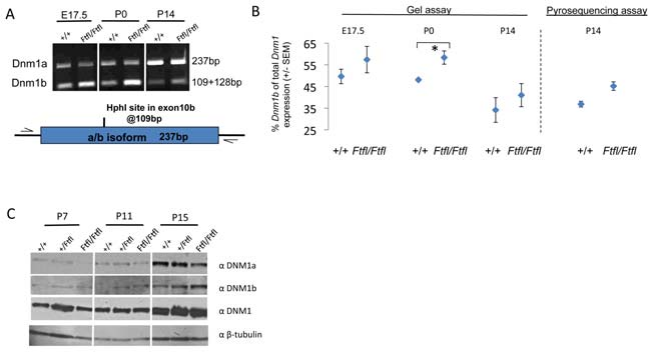
Developmental expression patterns of dynamin-1 isoforms. (A) mRNA expression levels of Dnm1 isoforms during development in wildtype and mutant animals. The variant isoform region is amplified with common primers and the two transcripts are distinguished by a diagnostic *Hph*I restriction enzyme site specific for the b isoform. The two bands representing the *Dnm1b* transcript cDNA run as one band and lower on the gel than the *Dnm1a* transcript cDNA. Note that the 1a isoform becomes increasingly upregulated during development, while the b isoform is down regulated, however this ratio of a to b transcripts is skewed in the mutant animals. Below the gel is a schematic outlining the basis of the assay. Additional replicates are shown in Figure S2. (B) *Left*, quantification of *Dnm1b* mRNA expression as assayed by PCR and visualized on agarose gels, relative to total *Dnm1* mRNA in whole brain of E17.5, P0, and P14 wildtype and mutant brains. Data are expressed as the mean (± SEM) relative proportion of *Dnm1b* to *Dnm1* mRNA in the total cleaved PCR product. *P<0.05, Student's t-test. *Right*, relative quantification of isoform transcripts by pyrosequencing. cDNA from P14 wildtype or homozygous fitful brains was amplified by PCR, and subsequently analyzed by pyrosequencing and quantitated for *Dnm1b* levels relative to *Dnm1*; n = 3 for each set of cDNA. (C) Protein levels of Dnm1 isoforms during development in wildtype and mutant animals. Custom made antibodies that distinguish DNM1 exon10a and exon10b middle domains were used to assay isoform protein levels in brain extracts of mice during development. Note the decrease in DNM1A in homozygous fitful mice compared to wildtype at all three ages examined (P7, P11, P15). Overall DNM1 protein levels were detected with a commercial antibody against full length DNM1. For a loading control, β-tubulin levels were detected. Significant difference in levels of DNM1b in mutant (Least square regression analysis; P<0.05).

Dynamin-1 increases in abundance during early postnatal brain development [9], [12]. We examined the expression of *Dnm1a*x and *Dnm1b*x during brain development in mutants. cDNA amplification using a single set of primers for both isoforms, followed by restriction with *Hph*I which has a recognition site present only in exon 10b, allowed for direct visualization of the relative expression of exon 10a and exon 10b containing isoforms. From E17.5 through P14, the overall increase in *Dnm1* is due to isoforms containing exon 10a, while isoforms containing exon 10b either remain constant or diminish slightly with age (Figure 4A). Interestingly, in homozygous mutant, there was a delay in this shift; isoforms containing exon 10b remained upregulated longer (compare at P0) during development. This was significant as P0 through P14 is the period of extensive synaptic maturation and regulated endocytosis. Quantification of exon 10b-containing isoforms relative to the total amount of *Dnm1* mRNA in whole brain was calculated and showed a clear trend suggesting that *Dnm1b*x is more prominent in mutants than wildtype throughout development (Figure 4B, left panel). This shift was confirmed by quantification of relative isoform transcript levels using pyrosequencing (Figure 4B, right panel) and did not result in a significant change in overall *Dnm1* transcript abundance.

The alteration in isoform expression was confirmed and extended at the protein level using isoform-specific antibodies (see Figure S3 for details). Figure 4C shows one of three replicate western blots demonstrating a decrease in protein containing exon 10a peptides (DNM1a) at each age examined in mutants as compared to wildtype. There is a parallel increase corresponding to exon 10b containing peptides (DNM1b). Least square regression analysis with age, genotype and replicates as independent variables revealed a significant (p<0.05) increase in the expression of DNM1b in mutant animals after two weeks of age. These results suggest the possibility that altered dynamin-1 isoform composition during this important period of synaptic maturation may contribute to the disease phenotypes.

### DNM1 protein assembly defect in fitful mice

**Figure 5:**
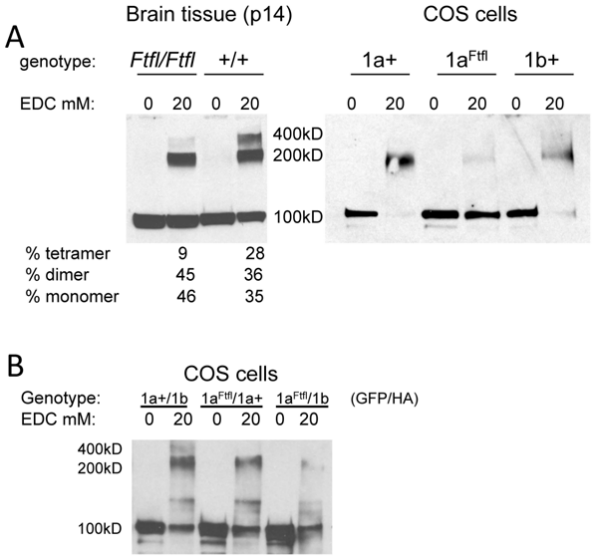
DNM1a^Ftfl^ is defective in higher order homo-oligomerization. (A) *Left panel*, protein extract from P14 whole brain tissue of homozygous fitful and wildtype littermates incubated with 0 or 20mM EDC cross-linker and hybridized with anti-dynamin-1 antibody. Monomers migrate at 100kD, dimers at 200kD and the tetramers are at 400kD. This assay was performed over three separate times with different samples each time; a representative blot with corresponding percentages is shown. Mean densities (± 1SD) from all experiments are: wildtype 28.75±8.24 (monomer), 29.67±13.9 (dimer), 43.9±8.5 (tetramer); mutant 44±10.7 (monomer), 39.5±12.2 (dimer), 23±13.5 (tetramer) *Right panel*, COS-7 cells transfected with DNM1-GFP constructs show differences in dimerization. (B) COS-7 cells doubly transfected with DNM1-GFP and DNM1-HA constructs show isoform heterodimerization. Protein extracts from cells were incubated with 0 or 20mM EDC and analyzed by Western blot. Blots were hybridized with anti-GFP antibody, stripped of antibody and then re-hybridized with anti-HA antibody in order to ascertain the presence of each construct in the dimers. A representative blot hybridized with anti-GFP antibody is shown.

The fitful mutation resides within the “middle” domain of dynamin (Figure 2A), previously shown to be required for self-assembly into dimers and further higher-order assembly into tetrameric structures [18]–[20]. We examined the ability of DNM1^Ftfl^ to properly assemble into dimers and higher order oligomers using protein extracts from normal and fitful brain. Protein extracts were incubated with a cross-linking agent, 1-Ethyl-3-[3-dimethylaminopropyl]carbodiimide hydrochloride (EDC), of zero length, to bind proteins intimately associated in dimer or tetramer formation [33]. Equal amounts of extract were treated with or without EDC and separated by SDS-PAGE. Protein from wildtype brains exhibited the ability to form dimers and tetramers in the presence of cross-linker (Figure 5A, left panel). Over half the DNM1 protein detected was assembled, with 28% in tetrameric form and 36% in dimeric form. While the homozygous fitful brain extract appeared to contain DNM1 dimers upon the addition of EDC, the amount of higher order structures (tetramers in this assay) was reduced with only 9% of detected DNM1 in tetrameric form (Figure 5A, left). This result indicates a defect in the ability of DNM1A^Ftfl^ to form multimeric DNM1 complexes.

The dimer band in the mutant extracts, which was 45% of the DNM1 signal, was possibly due to the assembly of normal DNM1b. To explore this further, we analyzed the ability of exogenous DNM1 isoforms to self-assemble in cells. We made isoform-specific constructs that express GFP fused to the C-terminal of DNM1. Equal amounts of each GFP-tagged DNM1 isoform construct was transfected individually into COS-7 cells. Both exogenous wildtype proteins, DNM1a and DNM1b, formed dimers efficiently (Figure 5A, right panel). This dimerization was accompanied by a corresponding reduction in monomer amount. Notably, mutant DNM1a^Ftfl^ did not form dimers efficiently and remained mostly monomeric in COS-7 cells (Figure 5A, right panel). These results confirm that DNM1a^Ftfl^ is defective in self-assembly.

To determine whether the mutant monomer can assemble with wildtype monomers to form heterodimers, we doubly-transfected COS-7 cells with equal amounts of GFP- and HA- C-terminal tagged dynamin-1 constructs. Upon cross-linking and analysis with GFP or HA antibodies, we observed that the mutant DNM1a^Ftfl^ protein can dimerize with each wildtype isoform (Figure 5B), albeit to a lesser extent than wildtype DNM1a.

### DNM1A^Ftfl^ interferes with endocytic trafficking in cells

Dynamins are catalytic for endocytosis. Extensive previous studies introducing various dynamin mutant constructs into mammalian cells have demonstrated that overexpression of mutant dynamin blocks endocytosis and causes an accumulation of endocytic intermediates [32]. Several of these mutations (e.g. K44A) are dominant-negative, binding to endogenous dynamin and preventing it from properly functioning [4], [16]. Specifically, dynamin GTPase domain mutations that inhibit endocytosis when expressed in COS-7 cells have a reticular type of localization as opposed to the more diffuse cytoplasmic localization of wildtype dynamin [34]. In prior studies to more closely examine these mutants by electron microscopy, the cells expressing mutant dynamin were observed to contain dynamin-coated plasma membrane tubules that had not undergone fission [34].

**Figure 6:**
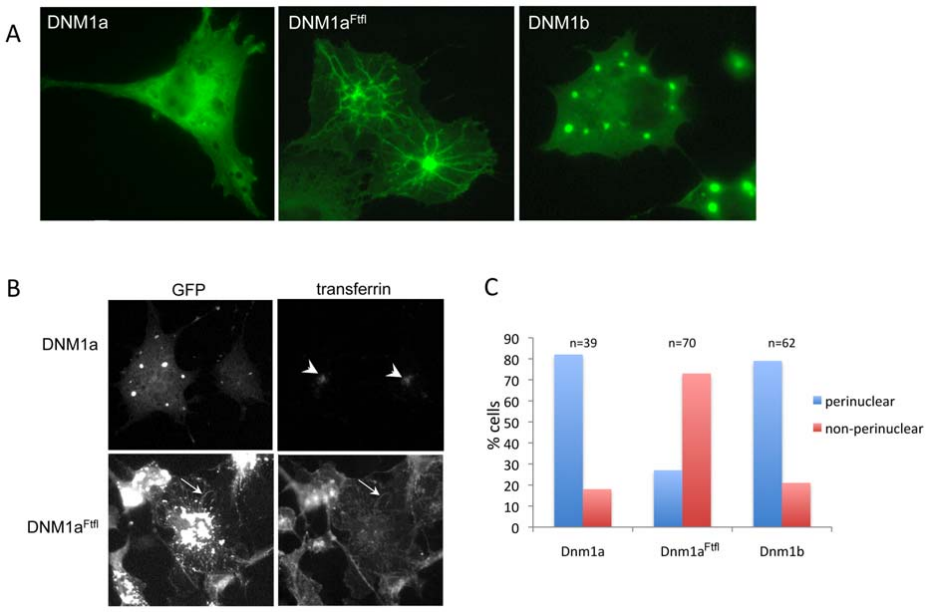
DNM1 localization and endocytosis in COS-7 cells. (A) DNM1 isoform localization in COS-7 cells over-expressing DNM1 isoform constructs. Left is wildtype, middle is DNM1a^Ftfl^ and right is DNM1b. Note the tubulation in the mutant expressing cells. (B) Transferrin endocytosis and localization in COS-7 cells overexpressing DNM1 isoform constructs. Left panels show DNM1 GFP fluorescence in COS-7 cells containing wildtype or DNM1a^Ftfl^; right panels show TRITC-transferrin uptake and localization in COS-7 cells containing wildtype or DNM1a^Ftfl^ -GFP. The arrowheads in the top right picture indicate the normal perinuclear accumulation of transferrin. The arrows in the bottom right picture indicate one point of co-localization between DNM1a^Ftfl^ and transferrin. (C) Quantification of transferrin localization in COS-7 cells overexpressing DNM1 isoform constructs. Transfected cells were determined to have transferrin localized to the region adjacent to the nucleus in a manner similar to non-transfected cells or to have not taken up transferrin at all or to have an abnormal localization of transferrin.

To examine the cellular localization of DNM1a^Ftfl^, we expressed GFP-tagged DNM1 isoform constructs in mammalian COS-7 cells. Wildtype DNM1a-GFP and DNM1b-GFP exhibited localization patterns similar to each other, characterized by a diffuse cytosolic distribution with bright puncta in the more highly expressing cells. However, in cells expressing DNM1a^Ftfl^-GFP the pattern was strikingly different. Notably, many of the transfected cells were observed to have fluorescent tubular networks very similar to the structures observed by others previously (see Figure 6A; [34]).

In order to determine whether DNM1a^Ftfl^ interferes with endocytosis, transfected COS-7 cells were tested for uptake of fluorescently labeled transferrin. Transferrin uptake was observed in cells expressing wildtype DNM1a (Figure 6B) and wildtype DNM1b (data not shown), but cells expressing mutant DNM1a^Ftfl^ were deficient in general transferrin uptake as characterized by the lack of perinuclear transferrin localization (Figure 6B). Overall, 82% and 79% of cells transfected with the wildtype DNM1a and Dnm1b, respectively, were observed to have taken up and properly localized the transferrin. However, only 27% of cells transfected with DNM1a^Ftfl^ had taken up transferrin; 73% either had no transferrin visible or it was mislocalized (Figure 6C), but often exhibited a notable co-localization with DNM1a^Ftfl^ at the tubular structures (Figure 6B, arrow). This observation is in agreement with the work carried out with dynamin GTPase mutants previously noted in which further investigation revealed transferrin-rich membrane invaginations continuous with the plasma membrane [34].

These results suggest that unlike wildtype DNM1a, DNM1a^Ftfl^ cannot support endocytosis in COS-7 cells. Furthermore, DNM1a^Ftfl^ seems to act in a dominant-negative manner, interfering with the function of endogenous DNM2 - the dynamin that normally carries out these functions in COS-7 cells - similar to previously described dynamin GTPase mutants that are not able to catalyze the fission of membrane and inhibit endocytosis [34]. Dominant-negative effects are currently thought to be due to hetero-oligomerization of the mutant oligomer with endogenous dynamin oligomers, perturbing normal function.

### Prolonged inhibitory synaptic activity in cerebral cortex slices

**Figure 7:**
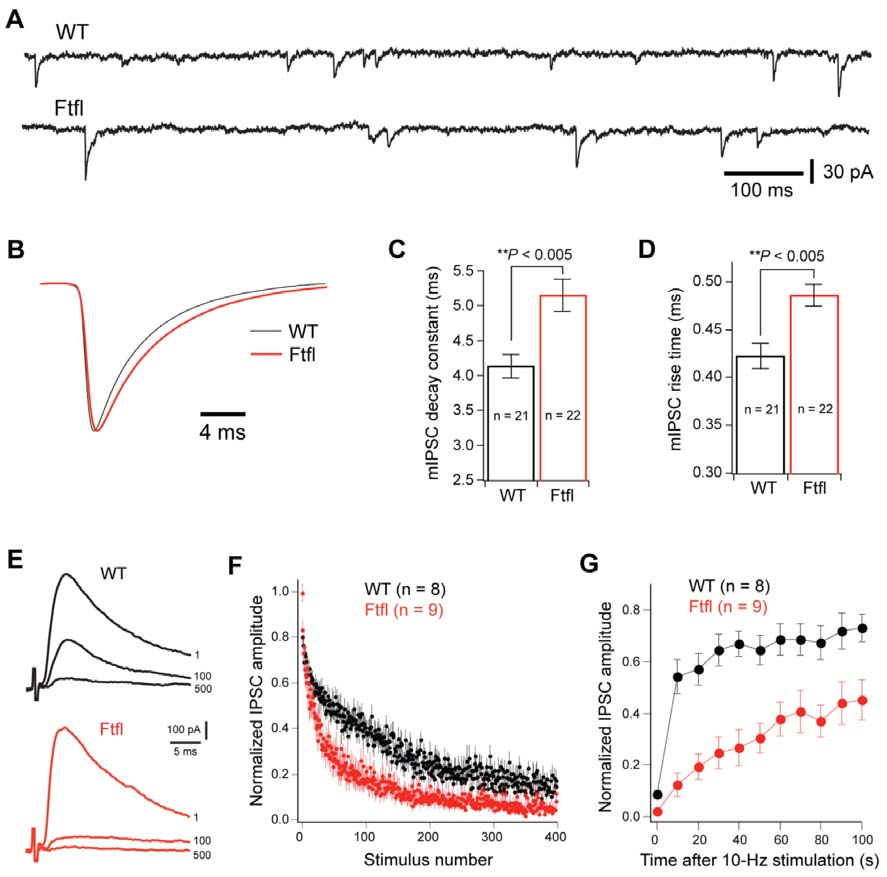
Altered GABAergic transmission in cortical neurons of fitful mice. (A) Samples of GABAergic mIPSCs recorded from a wildtype (upper trace) and a fitful (lower trace) layer V pyramidal neuron at P14 in the presence of DNQX, kynurenic acid, and TTX. The patch pipettes contained 130 mM KCl, and holding potential was at −70 mV. (B) Averaged mIPSCs from 21 wildtype cells (grey) and 22 fitful cells (red) with normalized peaks. (C,D) Histograms of the decay constant (C) and rise time (D) of mIPSCs for wildtype and fitful cells. The mean decay time constant was 5.2±0.2 ms (n = 22) for mutant cells, and 4.1±0.2 ms (n = 21) for wildtype (*p*<0.005, Mann-Whitney test). (E) Samples of evoked GABAergic IPSCs in response to 10-Hz stimulation recorded from a wildtype (upper black traces) and a fitful (lower red traces) cell at P14 in the presence of DNQX and kynurenic acid. For both cells, the three traces were responses to the first, 100^th^, and 500^th^ stimulus. The patch pipettes contained 110 mM cesium methylsulfate, and holding potential was at 0 mV. A pair of twisted microwire was placed in layer V to evoke monosynaptic IPSCs. (F) Plots of evoked IPSCs in response to 400 stimuli at 10 Hz. For each cell, peak amplitudes of IPSCs were normalized to that of the baseline response. (G) Recovery after 1000 stimuli at 10 Hz. IPSCs were measured every 10 s after the 10-Hz stimulation. Peak amplitudes of IPSCs were normalized to that of the baseline response before the 10-Hz train stimulation.

We examined synaptic properties of cortical neurons in acute slices for evidence of abnormalities in fitful homozygotes. We focused on GABAergic transmission because recent studies using neuronal cell culture demonstrated that the loss of *Dnm1* preferentially affects inhibition [9]. First, we recorded quantal GABAergic IPSCs (mIPSCs) in layer V pyramidal neurons in the somatosensory cortex at P14–15. Cells with comparable series resistance were analyzed (9.3±0.5 MW, n = 21 cells from three wildtype mice; 9.3±0.5 MW, n = 22 cells from three mutant mice). Samples of GABAergic mIPSCs recorded from a wildtype (upper trace) and a fitful (lower trace) layer V pyramidal neuron are shown in Figure 7A. Fitful neurons demonstrated more prolonged quantal events than those in wildtype mice (Figure 7B). The mean decay time constant was 5.2±0.2 ms (n = 22) for mutant cells, and 4.1±0.2 ms (n = 21) for wildtype (*p*<0.005, Mann-Whitney test). Quantal events in mutant also had slower rise time. The mean rise time (from 20 to 80% of the peak) was 0.49±0.01 ms for mutant, and 0.42±0.01 ms for wildtype, which was significantly different. There were no significant differences between mutant and wildtype in the frequency (10.8±0.8 Hz vs. 10.9±0.8 Hz; p>0.8) or peak amplitude of mIPSCs (42.9±3.8 pA vs. 34.5±3.2 pA; *p*>0.06; data not shown).

To determine whether the fitful mutation leads to a deficit in vesicle membrane recycling, we recorded monosynaptic GABAergic responses by applying current pulses through stimulation electrodes placed in layer V. There was no significant difference between mutant and wildtype cells in the maximum IPSC (1254±113 pA, n = ten cells from three mutant mice; 1209±132 pA, n = ten cells from three wildtype mice; *p*>0.5; data not shown). Trains of 10-Hz stimulation were applied at intensities that achieve 40 to 60% of the maximum response. As illustrated in Figure 7E and 7F, the amplitude of IPSCs decreased more rapidly in mutant than wildtype cells during 10-Hz stimulations. The recovery was tested by applying stimuli at 0.1 Hz immediately following 1000 stimuli at 10 Hz. The recovery of IPSCs was much slower in mutant than wildtype cells (Figure 7G; n = nine cells from two mutant mice; n = eight cells from two wildtype mice). The depression and the reduced rate of recovery in IPSC amplitude were affected in fitful, similar to the results in *Dnm1* null primary neuron culture [9]. These differences in evoked GABAergic response are consistent with a likely deficit in vesicle membrane recycling in mutant cells and suggests that the mutation in dynamin-1 interferes with sustained evoked release.

## Discussion

Fitful mutant mice reveal intriguing differences between underlying dynamin-1 mutations and the respective phenotypes. Alternative splicing in the molecular assembly-associated domain of dynamin-1 appears to play a key role in these phenotypes and in the proper function of dynamin-1. Fitful mice have a complex neurological disorder in which epilepsy is a major and consistent feature. Adult heterozygotes have recurrent limbic and tonic-clonic seizures, a modestly reduced seizure threshold, and a predisposition for becoming epileptic, but no other obvious phenotypic abnormalities. This dominant phenotype provides a new model for common idiopathic generalized epilepsy (IGE), for which few causative genes are known and progress is slowed by the complex genetic architecture underlying this disorder. In contrast, fitful homozygotes have multiple neurological deficits by three weeks of age, including delayed growth, cerebellar ataxia, hearing loss, and severe, ultimately lethal, tonic-clonic seizures. Fitful creates an amino acid substitution in the highly conserved DNM1ax isoform encoded by *Dnm1* exon 10, leaving DNM1bx structurally intact. While DNM1a^Ftfl^ protein is expressed in mutant mice, it does not efficiently assemble into higher order oligomeric dynamin complexes. In addition, similar to previously described dominant-negative alleles of *Dnm1*, characterized only *in vitro* (e.g. K44A; [4]), DNM1a^Ftfl^ also interferes with normal endocytosis in COS-7 cells.

Our heterologous expression experiments provide insight into the biochemical consequences of the DNM1a^Ftfl^ mutation, with its dominant-negative effect on endocytosis evidenced by a lack of transferrin uptake and subsequent proper trafficking. We can envision three ways in which this effect is manifest. First, sequestration of interacting proteins necessary for endocytosis could reduce the pool available for endocytosis. Preliminarily, however, we have found no evidence for altered abundance of several known dynamin-associated proteins, such as amphiphysin I and auxilin, in fitful homozygotes (our unpublished results). Second, assembly of non-functional DNM2 and DNM1a^Ftfl^ heterodimers could inhibit proper DNM2 function. This remains a possibility as we find that DNM1a^Ftfl^ can interact with DNM2 to some extent, both in COS-7 cells and also in brain (unpublished data), however we find no evidence of colocalization by immunofluorescence (unpublished data). We do find evidence for heterodimerization of DNM1a^Ftfl^ with each of the wildtype dynamin-1 isoforms (Figure 5B), suggesting a dominant negative effect whereby mutant protein binds with wildtype protein resulting in non-functional heterodimers that cannot fission membrane.

Third, the accumulation of unassembled DNM1a^Ftfl^ at sites of fission could stall endocytosis. This is an attractive possibility for fitful mice. The appearance of tubules in cells that overexpress DNM1a^Ftfl^ is reminiscent of previous dynamin mutations [34] and narrow tubules were also observed in dynamin-1 null neurons [9]. While tubules may represent a buildup of invaginating membrane that cannot be fissioned, they could alternatively result from stalled clathrin-mediated endocytosis at the invagination stage (a stage at which dynamin is thought to be a checkpoint; [35]). Membrane invagination and fission are mediated by two separate mechanisms carried out by dynamin [7] and both assembly-independent GTPase and assembly-stimulated GTPase activities are needed to carry out these process [35]–[37]. The assembly/disassembly cycle is required for association and disassociation with the membrane. Self-assembly defective DNM1a^Ftfl^ would presumably not have the assembly-stimulated activity required for fission, but would retain the assembly-independent activity necessary for membrane invagination. Furthermore, unassembled dynamin has been proposed to be the molecular checkpoint during early stages of clathrin-mediated endocytosis, potentially allowing the abortion or progression of clathrin-coated particle (CCP) intermediates through maturation [35], [37]. Dynamin assembly is suggested to control the transition from early to late events in CCP maturation [37]. Such a model would imply that unassembled dynamin can monitor CCP formation and is a separate activity from membrane fission as catalyzed by assembled dynamin. Our data showing that unassembled DNM1a^Ftfl^ can tubulate membranes but cannot fission them are consistent with this hypothesis.

We speculate that the seizure phenotype in fitful but not in *Dnm1*-null heterozygotes may result from the unique presence of mutant DNM1a^Ftfl^ stalling such an endocytic checkpoint. This would also provide an explanation for the reduced phenotype severity of compound heterozygous mice as compared with homozygous fitful (e.g. Figure 3A); homozygous fitful would have up to twice the amount of abnormal protein, accumulating and exerting its negative effect with a quicker timecourse. However, even the compound heterozygotes ultimately succumb to lethal seizures. This dominant effect of DNM1a^Ftfl^ presumably leads to a lack of sufficient vesicles for extended inhibitory transmission - particularly problematic for tonically firing inhibitory synapses [9], [10].

The genetic and phenotypic differences between fitful and *Dnm1* null mice - in particular, epilepsy in fitful heterozygotes but not in *Dnm1* null mice of either genotype ([9]; P. de Camilli, personal comm.; our unpublished observations) – further suggests that DNM1a^Ftfl^ protein confers unique properties relevant for disease. We examined both spontaneous and evoked IPSCs in layer V pyramidal neurons in acute brain slices. The peak amplitude of spontaneous or mIPSCs was not significantly different between fitful mutant and wildtype mice. This is in contrast with the results obtained in *Dnm1*-null cortical cultures where *Dnm1*-null neurons show a large increase in the peak amplitude of mIPSCs [9]. The reason for this difference is unknown. *Dnm1*-null neurons have larger diameter synaptic vesicles, which may lead to an increase in quantal content. This increase in vesicle size observed in *Dnm1*-null neurons may be caused by direct or compensatory mechanisms. It is possible that the presence of the mutated dynamin-1 in fitful mice attenuates compensatory responses. Another possibility is that fitful causes changes in the cable (electrical) properties of neurons or the location of GABAergic synapses, so that recordings from the soma are unable to detect changes in mIPSCs at the dendrites. Indeed, the slower kinetics of mIPSCs observed in fiftful neurons is consistent with this possibility.

However, the evoked IPSC responses in fitful acute slice preparations were similar to those shown previously in *Dnm1*-null primary neurons [9], reflecting a loss-of-function. Compared with wildtype, both alleles confer a more rapid depression of evoked IPSCs in response to 10-Hz stimulation, and a much slower recovery from depression. These findings, together with recent results obtained using a selective blocker of dynamin [38], demonstrate a critical role of dynamin in synaptic vesicle recycling. During repetitive stimulation, neurotransmission relies on the rapid reuse of synaptic vesicles endocytosed in a dynamin-dependent manner [9], [38]. Without efficient recovery of these rapidly reused vesicles, there is very likely a frequency-dependent exhaustion of synaptic inhibition, such a seen in other models of generalized epilepsy [23], [24].

The inability of DNM1a^Ftfl^ to assemble efficiently into oligomers is a plausible explanation for the biochemical defect, but to fully understand the development of disease, it may be important to consider functional differences between the two isoforms. Despite the high degree of conservation, functional differences conferred by the respective protein isoforms have not been demonstrated *in vivo*, but insight has been gained from heterologous systems [39], [40]. The alternate splice in exon 10 that occurs in *Dnm1* and *Dnm2*, but not *Dnm3* or in the dynamin-related protein *Drp*, has been recognized previously. We observe that this diversification is present in all jawed vertebrates and is even present in sea lamprey - a primitive, jawless freshwater fish – which, despite having these alternate exons, unlike other vertebrates possesses only a single dynamin gene (Figure 2B). Furthermore, neighbor-joining best tree analysis suggests that invertebrate dynamins are closer to DNM1b (and DNM2), whereas DNM1a forms a more isolated phylogenetic group (Figure 2C). The divergent exon 10a may well be a specialization that is unique to vertebrates.

The developmental shift in isoform expression may also provide clues into function. DNM1b expression is highest during embryonic and early postnatal development and decreases with synaptogenesis as DNM1a expression increases (Figure 4A and 4C). This may be to accommodate changing requirements for endocytosis over the course of development. At the onset of synaptogenesis, basal endocytosis is down-regulated and stimulation-induced endocytosis takes over as the major form of SV recycling [41]. If one form of endocytosis relies more heavily on a specific DNM1 isoform, then the altered isoform expression in fitful may disrupt the changes in endocytosis necessary for maturation, resulting in a number of adverse consequences. Early endocytosis is required for many vital processes such as growth cone development, cellular migration, axonal arborization and dendritic branching. It is possible that the significantly stunted Purkinje cell arborization observed in the homozygous fitful mutants is one consequence of an early developmental abnormality. Alternatively, inappropriate expression of DNM1b in mature fitful neurons that should predominately express DNM1a, may adversely affect the kinetics of endocytosis, in a manner suggested above.

It is intriguing that a missense mutation in a discrete splice variant of *Dnm1* can lead to significant neurological disease phenotypes. We speculate that *Dnm1* isoforms confer specificity to both the developmental program of endocytosis and also to variant endocytic response to stimuli (e.g. clathrin-mediated *vs.* activity-dependent bulk endocytosis). In the future, studies using conditional isoform-specific expression will help to distinguish these possibilities.

## Materials and Methods

### Ethics statement

All animal procedures followed Association for Assessment and Accreditation of Laboratory Animal Care guidelines and were approved by institutional Animal Care and Use Committee.

### Mice

The fitful mice arose at The Jackson Laboratory (Bar Harbor, ME) as a spontaneous mutation on the C57BL/6J inbred strain in 2000. B6.129s1-Dnm1^tm1PDC^ mice were a gift from P. De Camilli [9]. To generate GFP mice used for cerebellar histology, fitful heterozygotes were mated to a B6 mouse strain carrying a transgene with the parvalbumin promoter fused to the GFP gene (strain # B20; [42]). All strains were housed in the Research Animal Facility at The Jackson Laboratory where animal procedures were approved by the ACUC.

### Genome scans

Two separate genome scans were used to map fitful to mouse Chromosome 2. To map the dominant seizure phenotype, B6-*Ftfl* heterozygotes were crossed to FVB/NJ mice. Two (of six total) resultant F_1_ hybrids were noted to have spontaneous limbic and tonic-clonic seizures after two months of age upon routine handling and weekly cage changes. The F_1_ hybrids were backcrossed to FVB/NJ mice, and the resultant N_2_ progeny were aged and observed at least weekly for seizures. Genomic tail DNA was prepared from 79 backcross mice, 22 of which showed at least two limbic and tonic-clonic behavioral seizures, and a genome scan was performed with seizure occurrence as a binary trait using microsatellite markers. The mutation was provisionally mapped to proximal Chromosome 2 and the critical interval for the recessive phenotype was ultimately refined to the genomic region between *D2Mit152* and *D2Mit203* after typing a total of 487 F_2_, F_3_ and F_4_ progeny.

Independently, the recessive ataxia and early onset seizure phenotypes were mapped after crossing B6-*Ftfl* heterozygotes to CAST/Ei, and subsequently crossing the F_1_ hybrids *inter se*. Provisional linkage to Chromosome 2 was established in 20 affected mice, and the critical interval for the recessive phenotype was ultimately refined to the genomic region between *D2Mit80* and *D2Mit72* after typing a total of 990 F_2_, F_3_ and F_4_ progeny.

### Genotyping the *Dnm1^Ftfl^* allele

Genotyping *Dnm1^Ftfl^* (fitful) was done by PCR using the primers: Dnm1 MwoI F2 (5′-CGGACGGGCCTCTTCACACCTG-3′) and Dnm1 MwoI R (5′-GCGGCCATACCTTTTCACTA-3′). The PCR product was digested for 2 hours at 60°C with the restriction enzyme *MwoI* (NEB). The digestion products were separated and visualized on a 4% Metaphor agarose (Lonza) gel by electrophoresis.

### Constructs

The *Dnm1ab*, *Dnm1a^Ftfl^b*, or *Dnm1bb* sequence was cloned into the pCMV-GFP and pCMV-HA vectors, in frame. The “b” alternative exon 22 was chosen because it is the most represented isoform found in Ensembl and the UCSC Genome Browser. This isoform was also the most abundant to clone from mouse cDNA. All constructs were confirmed by direct sequencing. Primers used to amplify *Dnm1* sequences were: Dnm1u (5′-CCATCGATATGGGCAACCGCGGCATGG-3′); Dnm1d (5′-CCGCTCGAGGGGGTCACTGATAGTGATTC-3′).

### Cell culture

COS-7 cells were maintained in supplemented Dulbecco's modified Eagle's medium (DMEM; 10% fetal bovine serum, 30U/ml penicillin, 30µg/ml streptomycin) at 37°C in a 5% CO_2_ humidified atmosphere. Cells were split twice a week.

### Transfections

Transient transfection of COS-7 cells was performed with 1–2µg of DNA/well in 6-well culture dishes using the Lipofectamine Plus Reagent (Invitrogen) according to the manufacturer's protocol.

### Endocytosis assays

To assay endocytosis, cells transfected (on coverslips) with GFP tagged proteins were incubated in serum-free DMEM (Invitrogen) for 1 hour at 37°C followed by the addition of 25µg/ml AlexaFluor-555 conjugated transferrin (Invitrogen) for 15 minutes at 37°C. Cells were washed three times with PBS, and in some experiments, were incubated in an acidic solution (0.5M NaCl, 0.2M acetic acid in PBS; pH 3.0) for 4 minutes at 4°C to strip surface bound transferrin and washed immediately with PBS, fixed in 4% paraformaldehyde, 4% sucrose in PBS for 10 minutes at RT and mounted on slides with Gel/Mount (biomeda).

### Reverse-transcription PCR

Total RNA was prepared from brains of E17.5, P0 and P14 fitful homozygous and wildtype littermates with Trizol (Invitrogen) following the manufacturer's suggested conditions and protocol. RNA (2µg) was reverse transcribed with AMV reverse transcriptase (Promega). cDNA was diluted and amplified for 25 cycles at an annealing temperature of 55°C with the following pair of primers: Dnm1 ex9F (5′-GAACTGCGA AGGGAGATCAG-3′) and Dnm1 ex12R (5′-GGTCACAATTCGCTCCATCT-3′) corresponding to Dnm1 exon 9 forward and exon 12 reverse, respectively. The PCR amplifications from three pairs of age-matched mice were run in triplicate. The PCR products were digested with the *HphI* restriction enzyme (NEB) overnight at 37°C and examined on a 2% agarose gel.

### Pyrosequencing

Pyrosequencing of cDNA from wildtype, heterozygous and fitful P15 brains was carried out by the Transgenic Genotyping Services facility at The Jackson Laboratory. PCR amplification of *Dnm1* was done with an initial denaturation step of 94°C for 5 min, followed by 50 cycles of denaturation at 94°C for 20 s, annealing at 60°C for 10 s, and extension at 65°C for 30 s. Final termination of the elongation step was carried out at 65°C for 5 min. The sequences of all of the primers are listed below. The biotinylated PCR products were prepared for pyrosequencing analysis by the use of a Vacuum Pre Workstation (Biotage AB), and the sequencing reactions were carried out on a PSQ 96MA system (Biotage AB) as described by the manufacturer. Sequence analysis software (Biotage AB) was used for measurement of the peak heights. Primers:

pIMR220F1, AGCTATGCTATCAAAAATATCCA; pIMR221R1,Biotin ACCATGTCCACACACTTGA; pIMR222S1b, CTCTTTACCCCAGACATG; pIMR223S1a, CTCTTCACACCTGACCTC.

### Western blot

Protein extracts were made in IP lysis buffer (20mM Tris, 0.5mM EDTA, 100mM NaCl, 0.5% NP-40) with Complete-mini proteinase inhibitor mix (Roche) added fresh. Extracts were quantified using the Bradford reagent (Bio-Rad). Extracts (50–100µg protein) were diluted in Laemmli buffer, incubated at 95°C for 5 minutes, resolved by SDS-PAGE and transferred to nitrocellulose membrane. All membrane blotting steps were carried out in TBS plus Tween (TBST) with 5% non-fat dry milk. Blots were incubated at RT with primary antibody (for specific dilutions see below) for 1–2h, HRP-conjugated secondary antibody (1∶5000) for 1h and visualized with the ECL plus kit (VWR Scientific). Membranes were incubated with Restore Western blot stripping buffer (Fisher) at 37°C for 15 min while shaking to remove antibodies for subsequent hybridization. Primary antibodies used were dynamin-1 (1∶1000; Affinity BioReagents, PA1-660), dynamin-1 (1∶500; Chemicon, MAB5402), dynamin 2 (1∶200; Santa Cruz Biotechnology, sc-6400), dynamin 2 (1∶250; BD Biosciences, 61025), dynamin 3 (1∶1000; Affinity BioReagents, PA1-662), dynamin-1a (1∶500; Affinity BioReagents), dynamin-1b (1∶300; Affinity BioReagents). Secondary antibodies used were HRP anti-goat (1∶5000; Santa Cruz Biotechnology), HRP anti-mouse (1∶5000; Thermo Scientific), HRP anti-rabbit (1∶5000; BioRad) and HRP anti-chicken (1∶5000; Santa Cruz).

### Oligomerization

Protein extracts made with IP lysis buffer were used for oligomerization assays. 50 or 100µg of protein was incubated with 0 or 20mM EDC (1-Ethyl-3-[3-dimethylaminopropyl]carbodiimide Hydrochloride; Pierce) in a final constant volume between samples for 45 minutes at RT in the dark. Samples were then diluted in Laemmli buffer, incubated at 95°C for 5 minutes, resolved by SDS-PAGE, transferred to nitrocellulose membrane and processed as for Western blots (see above).

### Immunofluorescence microscopy

For cryosection staining, sections were fixed with 4% paraformaldehyde, 4% sucrose in PBS for 15 minutes at RT. Slides were washed 3 times with PBS and blocked and permeabilized in PBS, 3% BSA and 0.5% Triton-X. The slides were incubated with primary antibody (anti-Calbindin; Swant, CB38) diluted in block overnight at 4°C, washed three times with PBS-T and incubated with secondary antibodies diluted in block for 1 hour at RT. Cells were washed three times with PBS, stained for 5 minutes with DAPI, washed and mounted with GelMount (Biomeda).

To analyze the GFP-Purkinje cells, brains from F2 homozygous fitful and controls were dissected at 17 days of age for fixation in Z-fix buffer (Anatech, Battle Creek, MI, USA) for 3–4 h. Vibrotome sagittal sections (60–100 µm) were mounted onto slides with Clear-mount (Zymed, San Francisco, CA, USA). The sections were viewed using a Leica SP5 AOBS spectral confocal microscope, with a 63×, 1.3 NA glycerol immersion objective. Excitation was at 488 nm, with emission collection optimized to detect green fluorescence.

### Electroconvulsive testing

For electroconvulsive testing, we followed procedures described previously with some modifications [43], [44]. Briefly, mice were restrained, a drop of anesthetic containing 0.5% tetracaine and 0.9% NaCl was placed onto each eye and a fixed electrical current was applied via silver transcorneal electrodes using an electroconvulsive stimulator (Ugo Basile model 7801). For acute electroconvulsive threshold ECT, the stimulator was set to produce rectangular wave pulses with the following parameters: 299 Hz, 0.2 s duration, 1.6 ms pulse width. The threshold to a minimal clonic forebrain seizure was determined by testing individual mice approximately daily until the endpoint was observed and group means were calculated. For determining the latency to kindling, the same electrodes were used with parameters that were estimated to yield a similar integrated RMS as described previously by others using sinusoidal waveforms [45]; 299 Hz, 3.0 s duration, 0.2 ms pulse width, 4.5 mA. Individual mice were challenged once daily until a partial seizure was observed, and group means were calculated to determine mean latency.

### Auditory brainstem response

Experiments were performed as described in [46], complied with national animal care guidelines and were approved by the University of Goettingen Board for animal welfare and the animal welfare office of the state of Lower Saxony. In brief, animals were anaesthetized intraperitoneally with a combination of ketamine (125 mg/kg) and xylazin (2.5 mg/kg) and the core temperature was maintained constant at 37°C. For stimulus generation, presentation and data acquisition we used the TDT III Systems (Tucker-Davis-Technologies, Ft Lauderdale, FL) run by BioSig32 software (TDT). Tone bursts (4/8/12/16/24/32 kHz, 10 ms plateau, 1 ms cos^2^ rise/fall, calibrated and provided in dB SPL rms) were applied at 20 Hz in the free field ipsilaterally using a Monacor DT-119 (Monacor, Bremen, Germany). The difference potential between vertex and mastoid intradermal needles was amplified (5×10^4^-times), filtered (low pass: 4 kHz, high pass: 400 Hz) and sampled at a rate of 50 kHz for 20 ms, 2×2000 times to obtain two mean ABR traces for each sound intensity. Hearing threshold was determined with 10 dB precisions as the lowest stimulus intensity that evoked a reproducible response waveform in both traces, as judged by visual inspection.

### Patch-clamp electrophysiology

Acute brain slices were prepared from *Ftfl* homozygotes and wildtype littermates at P14 using methods described previously [47]. Briefly, mice were anesthetized with tribromoethanol (250 mg/kg, i.p.) and decapitated. Brains were quickly removed and transferred into ice-cold solution containing (in mM): 210 sucrose, 3.0 KCl, 1.0 CaCl2, 3.0 MgSO4, 1.0 NaH2PO4, 26 NaHCO3, 10 glucose, saturated with 95% O2 and 5% CO2. Coronal slices were cut at 300 µm on a vibratome (VT 1000s, Leica) and kept in artificial cerebral spinal fluid (ACSF) containing (in mM): 124 NaCl, 3.0 KCl, 1.5 CaCl2, 1.3 MgSO4, 1.0 NaH2PO4, 26 NaHCO3, and 20 glucose, saturated with 95% O2 and 5% CO2 at room temperature (21–23°C). Slices were allowed to recover for at least 1 hr before any recording. Recordings were made at 32–34°C using whole-cell patch-clamp techniques. Each slice was transferred to a submerge-type chamber where it was continuously exposed to ACSF heated to 32–34°C, saturated with 95% O2 and 5% CO2, and flowing at rate of 2.0±0.2 ml/min. Whole-cell patch clamp recordings were made at the soma of layer 5 pyramidal neurons of the somatosensory cortex using a 40× water immersion objective (40×/0.80W, Nikon) and infrared Nomarski optics. Patch pipettes were pulled from thick wall borosilicate glass (1.5/0.84 mm, WPI) on a horizontal puller (P-97, Sutter Instruments). Resistance of electrodes was between 2 and 4 MΩ. Liquid junction potential was not corrected. Seal resistance was greater than 2 GΩ. Recordings were made with a Multiclamp 700B amplifier (Molecular Devices). The series resistance (Rs), usually between 8 and 14 MΩ, was monitored throughout the recording, and data were discarded when Rs varied by 20% or more over the course of the recording. GABAergic IPSCs were selectively recorded by blocking ionotropic glutamate receptors with 20 µM DNQX (6,7-dinitro-quinoxaline-2,3-dione) and 1 mM kynurenic acid. For evoked IPSCs, the pipette solution contained (in mM): 110 Cs methylsulfate, 20 TEA-Cl, 15 CsCl, 4 ATP-Mg, 0.3 GTP-Na, 4 QX-314, 0.5 EGTA, and 20 HEPES (pH 7.2, 270–280 mOsm). A pair of twisted nichrome microwires (38 mm in diameter, A-M Systems) were placed in layer V about 200 mm away from the recorded neuron. IPSCs were evoked by current pulses (20–400 mA, 50 ms). For mIPSC recording, the pipette solution contained (in mM): 130 KCl, 4 ATP-Mg, 0.3 GTP-Na, 0.5 EGTA, and 20 HEPES (pH 7.3, 270–280 mOsm with sucrose). Quantal events were recorded in the presence of tetrodotoxin (TTX, 0.4 µM). DNQX and QX-314 were obtained from Tocris; all other chemicals were obtained from Sigma-Aldrich USA.

Experiments were conducted using the AxoGraph X program (AxoGraph Scientific) with a PowerMac G5 connected to an ITC-18 interface. Data were filtered at 4 kHz and digitized at 16 kHz. Data were analyzed using AxoGraph X and IgorPro (WaveMetrics). Quantal IPSCs were detected using variable amplitude template functions with the rise time set at 0.5 ms and decay times. The detection threshold was set at four times the standard deviation of baseline noise. At least 100 isolated events for each cell were aligned and averaged to give the mean response.

## Supporting Information

Figure S1 Distortion product otoacoustic emissions (DPOAE) in *Ftfl* and wildtype/heterozygous and mice. DPOAE at 2f1-f2 were recorded from fitful mice (open symbols, n = 14) and wildtype/heterozygous mice (filled symbols, n = 23). (A) No significant differences in DPOAE levels were observed when testing different primary tone frequencies at stimulus levels of 60dB. (B) No significant differences in amplitude growth functions at 16 kHz were observed. The flat lines represent the noise floor+2 SEM (obtained from frequencies neighboring 2f1-f2).
(0.27 MB TIF)

Figure S2 Developmental expression of dynamin-1 isoforms. Shown are three separate PCR amplifications of cDNA from wildtype and homozygous Fitful whole brains at the time points indicated above the gels. The variant isoform region is amplified with common primers and the two transcripts are distinguished by a diagnostic *HphI* restriction enzyme site specific for the b isoform. The two bands representing the *Dnm1b* transcript cDNA run as one band and lower on the gel than the *Dnm1a* transcript cDNA. The actin transcript is amplified as control for cDNA levels.
(0.28 MB TIF)

Figure S3 Isoform specific antibody production. Custom antibodies were produced by Affinity BioReagents “Antibody on Demand” production services. Protein sequences from the two alternative exon 10 alleles were used to design two peptide antigens for each region. The two Dnm1a specific peptides were used to immunize rabbits and the two Dnm1b specific peptides were used to immunize chickens. The serum was collected and affinity purified. The antibodies were first analyzed for their isoform specificity by western blots using protein extracts from Cos-7 cells containing isoform specific constructs.
(0.07 MB TIF)

Video S1 Phenotype of fitful homozygous mice. The fitful homozygotes show a sever ataxia which is characterized by a wobbly stance and uncoordinated movement. Seen in the video is a P14 fitful mutant on a FVB background.
(1.77 MB MPG)
