# Supplementary figures and images for: A Missense Mutation in a Highly Conserved Alternate Exon of Dynamin-1 Causes Epilepsy in Fitful Mice
Source: PLoS Genet. 2010 Aug 5;6(8):e1001046. doi: 10.1371/journal.pgen.1001046 (PMC2916854; doi:10.1371/journal.pgen.1001046)

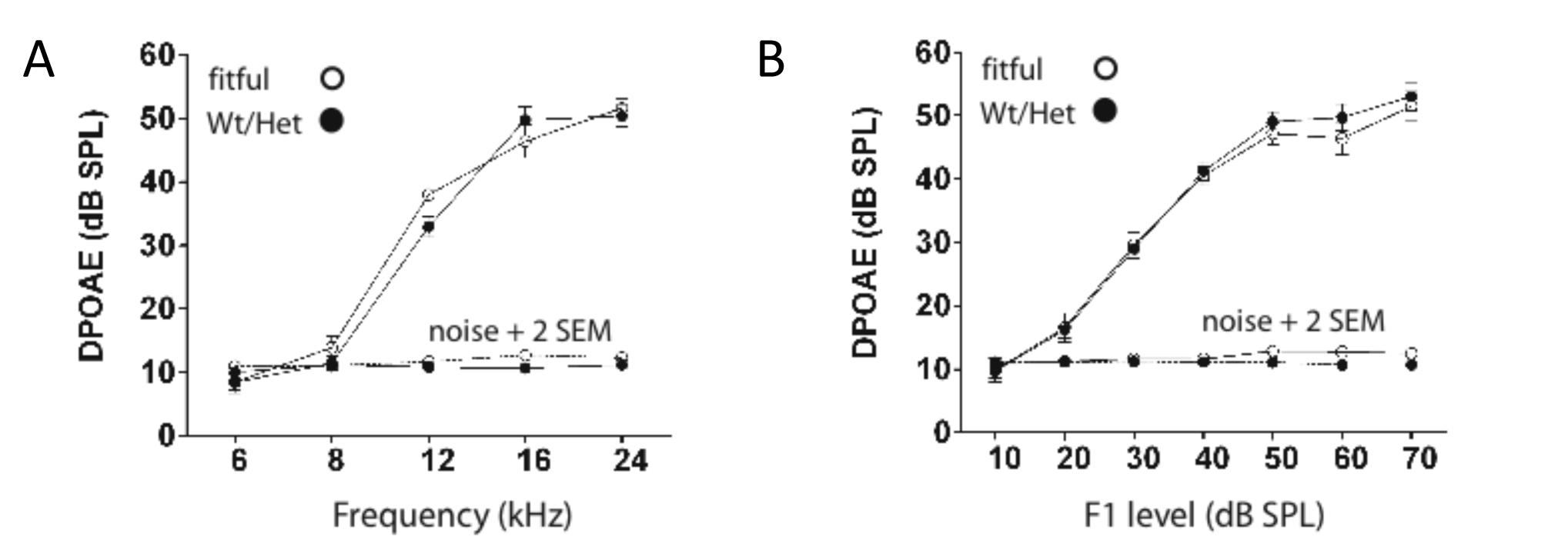

Supplement: Figure S1 — Distortion product otoacoustic emissions (DPOAE) in Ftfl and wildtype/heterozygous and mice. DPOAE at 2f1-f2 were recorded from fitful mice (open symbols, n = 14) and wildtype/heterozygous mice (filled symbols, n = 23). (A) No significant differences in DPOAE levels were observed when testing different primary tone frequencies at stimulus levels of 60dB. (B) No significant differences in amplitude growth functions at 16 kHz were observed. The flat lines represent the noise floor+2 SEM (obtained from frequencies neighboring 2f1-f2). (0.27 MB TIF) [file pgen.1001046.s001.tif]

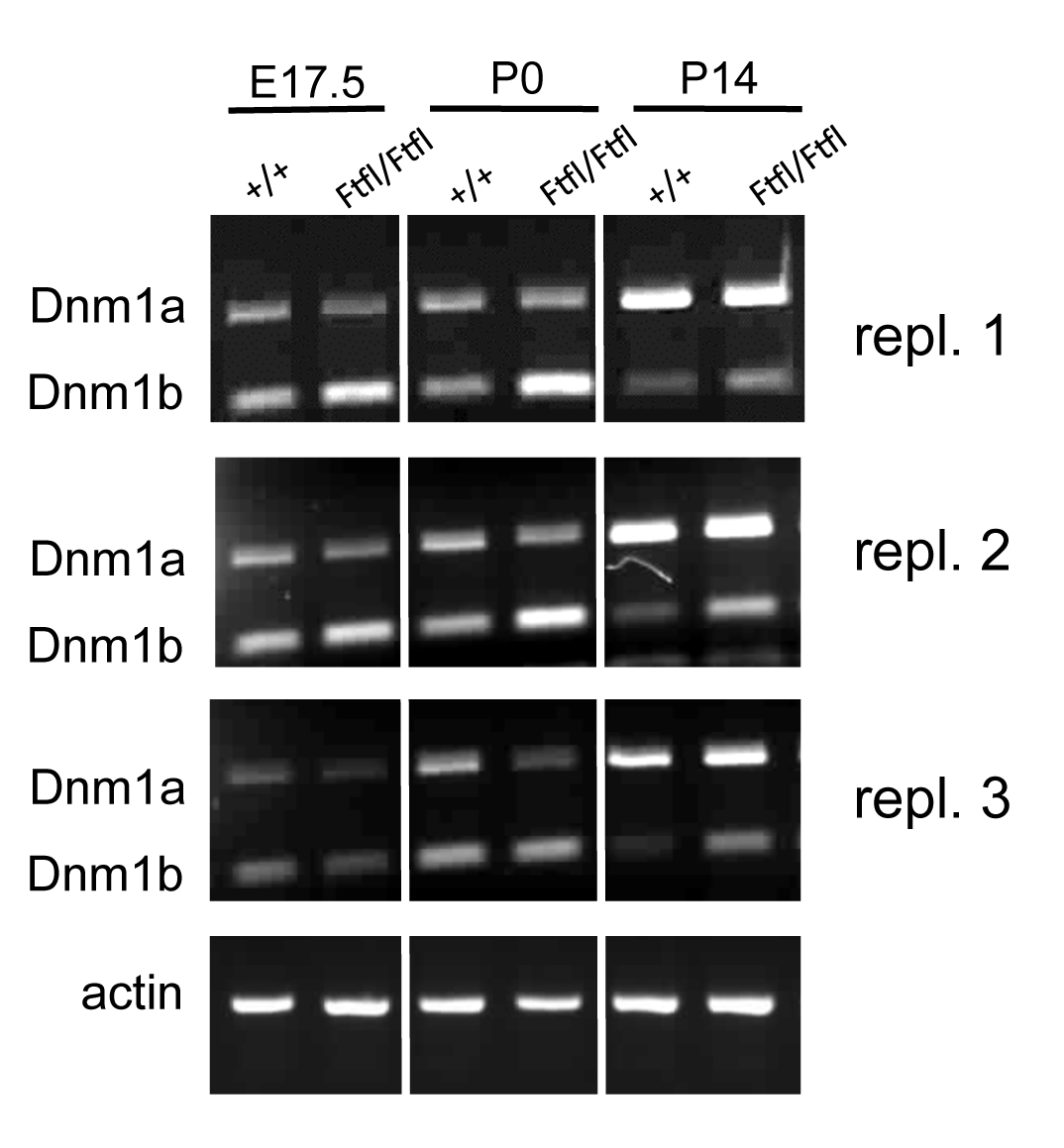

Supplement: Figure S2 — Developmental expression of dynamin-1 isoforms. Shown are three separate PCR amplifications of cDNA from wildtype and homozygous Fitful whole brains at the time points indicated above the gels. The variant isoform region is amplified with common primers and the two transcripts are distinguished by a diagnostic HphI restriction enzyme site specific for the b isoform. The two bands representing the Dnm1b transcript cDNA run as one band and lower on the gel than the Dnm1a transcript cDNA. The actin transcript is amplified as control for cDNA levels. (0.28 MB TIF) [file pgen.1001046.s002.tif]

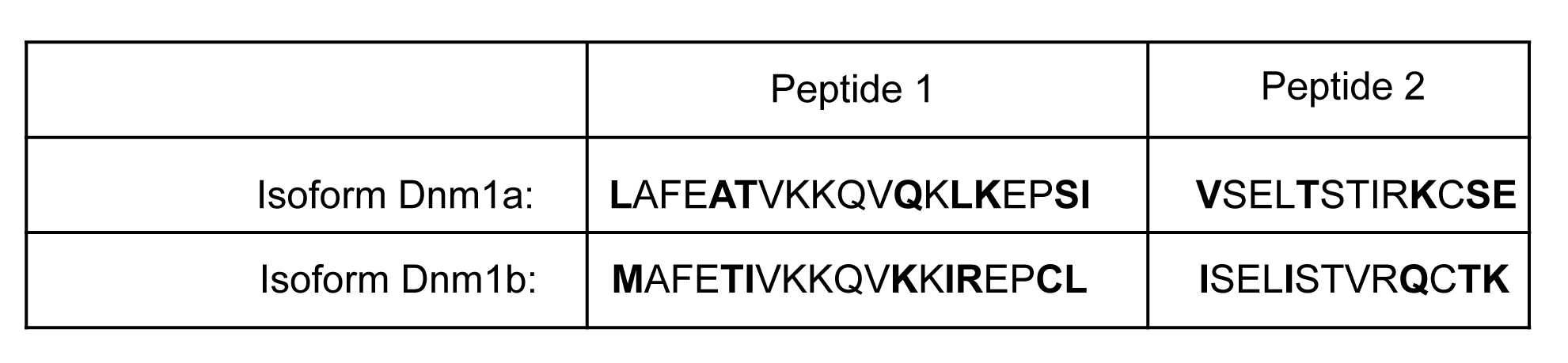

Supplement: Figure S3 — Isoform specific antibody production. Custom antibodies were produced by Affinity BioReagents “Antibody on Demand” production services. Protein sequences from the two alternative exon 10 alleles were used to design two peptide antigens for each region. The two Dnm1a specific peptides were used to immunize rabbits and the two Dnm1b specific peptides were used to immunize chickens. The serum was collected and affinity purified. The antibodies were first analyzed for their isoform specificity by western blots using protein extracts from Cos-7 cells containing isoform specific constructs. (0.07 MB TIF) [file pgen.1001046.s003.tif]
